# Dr. Andrew Kamerling

**Published:** 1918-03

**Authors:** 


**Dr. Andrew Kamerling,** who died in Buffalo, Dec. 6. 1917, was believed to be the last surviving member of the old Buffalo Medical College, class of '66. He was born in Deventer, Holland in 1840.

